# Adaptive trials, efficiency, and ethics

**DOI:** 10.1186/s12916-019-1437-z

**Published:** 2019-10-22

**Authors:** Spencer Phillips Hey

**Affiliations:** 1 Program on Regulation, Therapeutics, And Law (PORTAL), Division of Pharmacoepidemiology and Pharmacoeconomics, Department of Medicine, Brigham and Women’s Hospital and Harvard Medical School, Boston, MA USA; 2 Harvard Center for Bioethics, Harvard Medical School, 641 Huntington Avenue, Boston, MA 02115 USA

**Keywords:** Adaptive trials, Trial methodology, Research ethics, Clinical equipoise

## Background

Adaptive clinical trials employ a class of study designs that take advantage of the accumulating state of evidence in a trial to make midstream modifications to the trial’s structure or parameters. Under the right conditions, adaptive trials can be more efficient than traditional, non-adaptive designs. For example, they can allow several hypotheses to be tested using the same infrastructure, or fewer participants to be enrolled to achieve the same level of statistical power. But what are some of these ‘right’ conditions that make adaptive trials more efficient? This is the question asked by Wason et al. [1], who are concerned that the methodological literature may have overstated some of the advantages of adaptive designs, and overlooked some of the disadvantages.

### When are adaptive trials most useful?

Consider, for example, outcome-adaptive trials, in which the treatment arms or allocation ratios are modified based upon preliminary outcome data. Wason et al. observe that the potential gains in efficiency from these designs are only realizable if the preliminary, accumulating outcomes are (1) reliable predictors of the final outcomes, and (2) measured quickly, relative to the total, planned length of the trial. If the first of these conditions does not hold, then the trial may end up being misleading, because it has adapted to outcome data that are not, ultimately, informative. If the second condition does not hold, then the trial may be no more efficient than a non-adaptive design, because the data needed to modify the trial does not accumulate fast enough, relative to the speed of enrollment.

Beyond the methodological implications of adaptive trials, the article by Wason et al. also raises some interesting questions at the intersection of trial efficiency and ethics. The authors point out that some outcome-adaptive designs may be more attractive to patients, because as the trial evolves, it can minimize the number of patients allocated to intervention arms that are later found to be inferior. Difficulty in recruitment is one of the biggest reasons trials are terminated [2], and there is evidence to suggest that allocation to inferior treatment (e.g., a placebo arm) makes some patients more reluctant to participate in trials [3]. Therefore, if scientifically appropriate use of adaptive trials can also increase patients’ willingness to enroll, then – at least on its face – this looks like a clear win–win opportunity.

### Lingering ethical tensions

On closer inspection, however, there are some ethical tensions lurking beneath the idea that adaptive trials are (or should be) more attractive to patients. One of these tensions stems from the principle of beneficence, which requires a trial to have a favorable balance of risks and benefits [4]. However, the benefits in this ethical calculus cannot be the direct benefits to the participants from the experimental interventions, because these benefits are precisely what is being tested. The risks and burdens on the trial participants must therefore be offset by the benefits to future patients. Accordingly, the informed consent process for adaptive trials must make this ethical foundation clear: even if allocation is weighted in favor of the better-performing arms in the study, it does not necessarily mean that participation in the adaptive (rather than non-adaptive) trial is more likely to provide a direct therapeutic benefit [5].

A second ethical tension stems from the principle of clinical equipoise, which demands a genuine uncertainty within the expert community about the relative therapeutic merits across all arms in a controlled trial [6]. Two consequences of clinical equipoise are most relevant here: (1) patients should not be exposed to anything less than competent care; and (2) patients should not be systematically disadvantaged by their participation in the trial.

On the one hand, adaptive trials that drop arms, such as the Multi-Arm, Multi-Stage (MAMS) design [7], seem to operationalize clinical equipoise in an intuitive way. As soon as it is known that an intervention arm is ineffective or inferior (i.e., the arm no longer satisfies the conditions of clinical equipoise), then it is dropped from the trial. However, for adaptive trials that weight allocation in favor of better-performing arms, the implications of equipoise are less intuitive. As long as there is (and should still be) uncertainty about which arm is truly better, then there is no reason to think that patients are benefitting more or less by ending up in one arm or the other. In other words, because every arm must be consistent with clinical equipoise, patients are not advantaged or disadvantaged by their allocation [8].

## Conclusion

If the above analysis is correct, adaptive trials may not be the win–win opportunity that they first appear to be. Some of the reasons why adaptive trials seem more attractive for patients may be based on misconceptions about what makes clinical trials ethical. An ethical trial is one in which every patient—no matter their allocation—has their interests protected and can be assured of receiving competent care [9]. Therefore, the fact that an arm or the structure of a trial evolves over time should not be understood or advertised as offering an advantage to the participants. Instead, adaptive trials should be understood as offering advantages to the research system as a whole. In this respect, adaptive trials can still be a win-win. When used under the appropriate conditions, as Wason et al. help to elucidate, adaptive trials can resolve scientific uncertainties, while minimizing the total burden on patient populations.

## Data Availability

Not applicable.
